# Reflections on a rural road to family medicine

**DOI:** 10.4102/safp.v64i1.5594

**Published:** 2022-09-02

**Authors:** John-D K. Lotz

**Affiliations:** 1 Madwaleni Hospital, Eastern Cape Department of Health, Elliotdale, South Africa; 2 Department of Family Medicine and Rural Health, Faculty of Health Sciences, Walter Sisulu University, Mthatha, South Africa

## Introduction

Rurality is difficult to define, but marked by above-average levels of unemployment and poverty, poor infrastructure, lower proportions of healthcare workers and unequal access to basic services, including healthcare. Rural might also be defined as ‘that less popular place’, for patients and practitioners alike. Why then should family physicians seek training and employment in rural areas? This Next5 piece hopes to bring with it one rural perspective that offers three reasons: with a measure of presumptuous poetic licence, we might explore them as contextual, clinical and individual.

## Context

This perspective stems from completing training as a family physician (FP) at Madwaleni Hospital (MH) – a rural district hospital on the Wild Coast of the Eastern Cape province, South Africa. Existing in the midst of the complex environment that is public healthcare in rural South Africa, such facilities remain fragile. Like most, Madwaleni’s history is as sinuous as the surrounding gravel roads. In its most recent chapter, it has seen a steady gain in momentum as a core group of clinicians have been able to find a measure of continuity with the support of a committed and evolving clinical team, but attrition remains a constant threat. Staff retention is notoriously difficult in rural settings.

My wife and I joined this growing team as young medical officers in 2014 and have found ourselves thus far retained. Our experience has been a tension between rural challenges, outweighed by unique rewards. Living in this setting is an inescapable and humbling reminder of the difficulties that rural communities face, while sharing work and life with a close-knit community of colleagues and friends here at the hospital has been a formative process in our lives.

In 2017, Walter Sisulu University introduced a decentralised programme to train FPs in-context. By committing registrars to four years of training, attracting FPs as supervisors and growing a network of support, it suddenly added a strong cord to the ‘safety net’ that has sustained the growth experienced at Madwaleni and changed the clinical culture of the hospital.

## Clinical

The capacity for pursuing clinical excellence and developing services at MH has since grown exponentially. Registrars identify gaps in local clinical exposure and supplement training with short periods of ‘inreach’ at other facilities. While accommodating these periods of absence takes coordination, they are seminal to progress and strengthen collaboration.

Family physicians and registrars have been directly involved in re-establishing a regular outreach programme supporting primary health care facilities, implementing the widespread use of point-of-care ultrasound, driving the growth of surgical and anaesthetic capabilities, and in the establishment of a neonatal unit. Their influence has benefited staff by providing a supportive framework to help oppose the roots of systemic burnout, facilitate personal growth through mentoring, and allow opportunities for safe clinical skills advancement. A culture of ongoing quality improvement and research has been established.

The coronavirus disease 2019 pandemic then provided a litmus test for the preparation of locally trained FPs and registrars. Their direct involvement in managing patients, sensitive leadership of staff and the introduction of novel systems to navigate the emergent requirements that the pandemic demanded, all helped cushion the hospital in crisis. Some may question whether training in a particular setting is generalisable, but this highlights the importance of ensuring that all FPs qualify as flexible, lifelong learners with a dynamic range of skills that can be applied to any setting, or indeed pandemic.

## Individual

On a personal note, training as an FP in a rural setting has been immensely satisfying. It allowed for career progression while still being able to serve rural communities, and training provided the tools needed to effect more meaningful changes for a greater impact. Potential FPs should expect rural settings to be challenging, but inevitably ripe with opportunities to put these tools into practice. This encouragement might do well with a word of warning though: we may all be prone to a version of the ‘saviour mentality’, but this can be counterproductive!

## Conclusion

This perspective then supports the call to place at least one FP in every district hospital and community health centre in the country,^1^ especially in rural areas. Family physicians hold the potential to bring stability and equity to rural healthcare, and decentralised rural training programmes offer a key to making this possible. Where FP posts do not yet exist, tertiary or regional posts may be well repurposed and deployed to district level to facilitate this process. Finally, to extend an existing model,^2^ – recruiting candidates from rural communities for later inclusion in local FP programmes might present exciting prospects.

While far from an exhaustive justification for rural FP training and work, the hope is that this short piece might further invigorate the national FP narrative, and maybe even inspire a few rural adventures. After all, it is our rural communities who stand to benefit greatly therefrom.
